# Flow cytometry for rapid analysis of bacteriostatic versus bactericidal effects in *Legionella pneumophila* disinfection

**DOI:** 10.1007/s00216-025-06055-z

**Published:** 2025-08-14

**Authors:** Yiao Liang, Lena Heining, Martin Elsner, Michael Seidel

**Affiliations:** Institute of Water Chemistry, Chair of Analytical Chemistry and Water Chemistry, TUM School of Natural Sciences, Technical University of Munich, Lichtenbergstraße 4, 85748 Garching, Germany

**Keywords:** Flow cytometry, Intact cell counting, Cultivation-independent method, Rapid method, Sustainable biocide usage

## Abstract

Biocides, encompassing both oxidizing agents such as chlorine and non-oxidizing agents like quaternary ammonium compounds, are extensively employed to minimize the risk of *Legionella pneumophila* (*L. pneumophila*) outbreaks in evaporative cooling systems. Currently, biocide effectiveness to combat *L. pneumophila* is determined by cultivation test methods, which have the disadvantage of long testing times and fail to detect a viable but non-culturable status of bacteria. Cultivation-independent bacterial counting methods would, therefore, be superior for future analysis of biocide effects. Herein, we employed total cell counting and intact cell counting by flow cytometry (FCM) as rapid, cultivation-independent, and untargeted methods for effect-based analysis of biocide effectiveness. We used total cell counting and intact cell counting to detect DNA damage and membrane integrity changes in response to oxidizing sodium hypochlorite (NaClO) and non-oxidizing benzalkonium chloride (BAC). Biocide effect analysis over time was conducted on a dedicated cartridge-based flow cytometer for bacteria analysis, rqmicro.COUNT. The effects of NaClO and BAC on cell membranes and DNA could be well determined and distinguished. Process water contaminated by *L. pneumophila* was treated with different concentrations of NaClO and BAC, followed by regrowth experiments to determine bacteriostatic versus bactericidal effects. The results showed that a considerable reduction in intact cell count was related to the bacteriostatic effects of the treatment and the success of the disinfection of *L. pneumophila* according to the standard cultivation method. A significant decrease in total cell count indicating DNA damage was associated with bactericidal effects. Hence, we could show the potential of total cell counting and intact cell counting by FCM to be used as a rapid and effect-based analysis method for minimizing both health-related risks and biocide usage in process water.

**Graphical Abstract:**

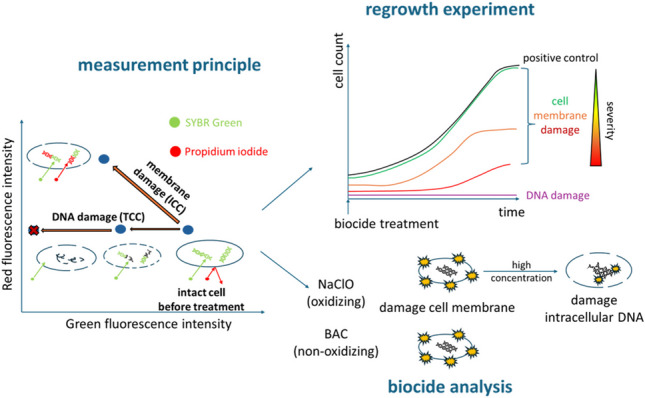

**Supplementary Information:**

The online version contains supplementary material available at 10.1007/s00216-025-06055-z.

## Introduction

The major purpose of biocide treatment in evaporative cooling systems and closed-circuit systems is to eliminate waterborne bacteria for avoiding outbreaks caused by pathogenic bacteria like *Legionella pneumophila* (*L. pneumophila*) [1, 2]. In the assessment of biocides for disinfection in evaporative cooling systems, the analysis of *L. pneumophila* by cultivation is still the gold standard (defined by EN 13623 [3]). Nevertheless, cultivation methods detect biocide effects on cultivability instead of specific cell components. A non-culturable bacterial cell can be a dead cell if it is severely and irreversibly damaged or in a viable but non-culturable status. Viable but non-culturable *L. pneumophila*, for instance, can rapidly resuscitate and grow under suitable conditions [4]. Cultivation methods fail to detect viable but non-culturable microorganisms, which can lead to severe hazard underestimation. Moreover, the long incubation times imply that cultivation methods are not well-suited for optimizing biocide dosage, as the results are available after 7–10 days.

Therefore, we tested the potential of intact cell counting and total cell counting by flow cytometry (FCM) to see if they are suitable analytical methods for rapid and effect-based biocide analysis. Total cell counting and intact cell counting are cultivation-independent and untargeted methods detecting the biocide effects on the whole microbial population. Intact cell counting by FCM has been used as a detection method for cell integrity changes during biocide treatments [5–7]. Total cell counting by FCM has recently been tested for detecting DNA damage in fungal spores during chlorine treatments [8].

Total cell counting uses cell-membrane-permeable fluorescence dyes like SYBR Green to stain all cells, including intact cells and cells with compromised cell membranes. Unlike SYBR Green, propidium iodide is a membrane-impermeable dye. An increased propidium iodide uptake therefore indicates altered cell membrane permeability, reflecting a loss of membrane integrity. For intact cell counting measurement, both SYBR Green and propidium iodide are used to differentiate between bacteria cells with intact and compromised cell membranes. Bacteria cells with compromised membranes are stained by both SYBR Green and propidium iodide, resulting in a much higher red fluorescence intensity. The cell count determined by total cell counting and intact cell counting are referred to as total cell count (TCC) and intact cell count (ICC), respectively.

**Fig. 1:**
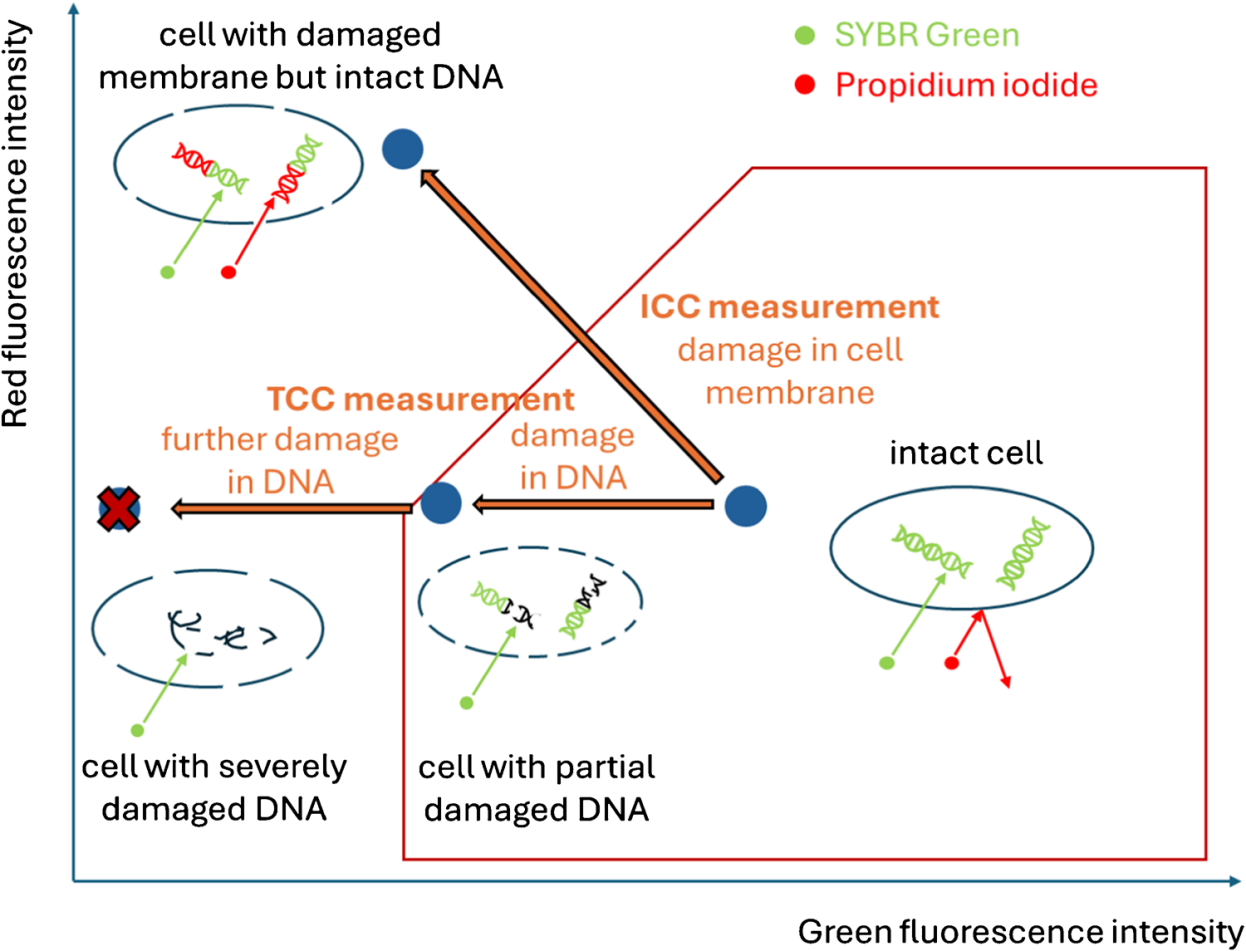
Schematic explanation for differentiating between DNA and membrane damage during biocide treatment using FCM-based analysis method

A typical approach for analyzing intact cell counting data is to plot the measured red fluorescence intensity against green fluorescence intensity for each cell, set a gate to separate intact cells from cells with compromised membranes, which exhibit higher red fluorescence intensity, and count the cells in the gate (shown in Fig. 1). For total cell counting data analysis, several previous studies used two distinct methodologies. One methodology assumed that damaged DNA could not be stained, and the fluorescence intensity should be reduced to a background level under detection threshold [7]. The other methodology assumed that the green fluorescence exhibited by damaged DNA should be reduced to a lower, yet still detectable, level [8]. We hypothesize that both patterns of reduction in green fluorescence intensity can indicate the presence of DNA damage, albeit reflecting different levels of severity. The experiments conducted by Phe et al. on isolated DNA samples provided us with a good basis for the following hypothesis [9]. As shown in Fig. 1, we assume that SYBR Green can still bind to double-stranded DNA with depredated nucleotide bases and lead to a lowered but detectable fluorescence intensity. If double-stranded DNA is more severely damaged and broken into single-stranded chains, the SYBR Green staining is negligible, reducing the fluorescence intensity to background level. In this work, we intend to optimize the total cell counting data analysis process for analyzing DNA damage and distinguishing between different levels of severity.

The commonly employed biocides in cooling systems can be divided into two classes: oxidizing biocides like sodium hypochlorite (NaClO) and non-oxidizing biocides like quaternary ammonium compounds. The efficiency of biocide treatment depends on various conditions, such as temperature, pH, organic compounds, biomass, microbial composition, and active concentration of the applied biocide. These known and unknown influential factors make the determination of biocide efficiency a challenging analytical task. In practice, biocides are typically overdosed to avoid the growth of *L. pneumophila* under varying operation conditions without analyzing their efficiency. Most guidelines recommend a shock biocide treatment at high concentrations (10–50 mg/L free chlorine) for disinfection and maintain a moderate active concentration, e.g., 2 mg/L free chlorine to avoid the growth of *L. pneumophila* [10–12]. Biocide overdosing leads to excessive chemical emissions from cooling systems. Chlorine can be rapidly degraded in water. However, overdosed chlorinated cooling water can create persistent organohalogen by-products like bromoform [13]. Some non-oxidizing biocides, like quaternary ammonium compounds, are widespread and persistent pollutants. Their emission can give rise to long-lasting contaminations of receiving waters and soil [14]. If biocide efficiency can be rapidly determined after biocide treatment, it is possible to conduct need-oriented biocide treatment and avoid such unnecessarily high biocide dosage.

To control bacterial growth, biocides have either bacteriostatic or bactericidal effects. Bacteriostatic effects mean that the biocide treatment prevents bacteria from growing. When the active concentration of biocide drops, bacteria can regrow, representing reversible biocide effects. In contrast, irreversible biocide effects are referred to as bactericidal effects. Here, bacteria are killed and cannot regrow even if the biocide is completely neutralized or rapidly degraded. In evaporative cooling systems, bactericidal or strong bacteriostatic effects are preferable, as they can control microbial growth and prevent an increase in pathogen concentrations for a relatively long term, reducing the frequency of biocide dosage. The number of components affected by biocide and the severity of the damage determine whether effects are bacteriostatic or bactericidal [15]. Depending on types and active concentrations, biocides can have effects on various cell components, such as cell membranes, enzymes, ribosomes, and nucleotides. For instance, the oxidizing biocide NaClO can alter the integrity of cell walls and membranes, reduce metabolic activity, and cause damage in intracellular DNA. The biocide effects of NaClO are strongly dependent on pH because pH influences the equilibrium HOCl and ^−^OCl, which have different membrane permeabilities and reactivities [16]. In comparison, the non-oxidizing biocide benzalkonium chloride (BAC), which is a quaternary ammonium compound, causes a loss in cell membrane integrity and has no significant effect on DNA [5]. According to mechanistic studies, irreversible biocide effects can be caused by cell component damage like intracellular DNA damage and changes to cytosolic pH after severe cell membrane damage [15]. As a result, it is possible to use suitable analytical methods based on biocide effects on cell membrane and DNA to estimate bacteriostatic versus bactericidal effects. Such an effect-based bioanalytical method would help to optimize biocide dosage for both microbial safety and sustainable biocide usage.

Previously, total cell counting and intact cell counting by FCM was primarily used for the principal mode-of-action study of biocides on isolated bacterial populations, for example, *Escherichia coli* [6, 8]. Nevertheless, this method is applicable for analyzing the complex microbial communities in process water and can offer an innovative solution for biocide effect analysis in engineered water systems. Currently, the choice of analytical methods is limited, typically between rapid but cost-intensive methods like qPCR and cost-effective cultivation methods that require long incubation times (7–10 days). Herein, we intended to test if total cell counting and intact cell counting by FCM, which is a rapid and affordable method (30 min, €5 per measurement), can provide non-specific proxies for pathogen disinfection. Furthermore, the results of total cell counting and intact cell counting can additionally correlate with the bacteriostatic or bactericidal effects of a biocide treatment, which is related to the biofilm formation. In this work, we employed total cell counting and intact cell counting by FCM for biocide analysis in evaporative cooling systems. To achieve that, we tested the cartridge-based flow cytometer rqmicro.COUNT, which was developed for bacteria analysis in process water and drinking water. After staining, cells pass one by one through a microfluidic channel in the cartridge. Their green and red fluorescence signals are excited by a laser and detected by sensors. Using this flow cytometer, quantifying *L. pneumophila* in 100-mL process water samples was possible in around 2 h, considering the time for microfiltration with immunomagnetic separation and flow cytometry [17]. The experiments were conducted on a stable but unique population of bacteria in process water. To prepare process water, we employed a specifically designed lab-scale plastic reactor, which simulates process water circulation in evaporative cooling systems and enables the growth of the microbial population under the defined conditions monitored by a sensor system. The applicability of total cell counting and intact cell counting on rqmicro.COUNT was tested for the oxidizing biocide NaClO and the non-oxidizing biocide BAC. We conducted biocide treatments on process water with spiked *L. pneumophila* and correlated the biocide effects on the whole microbial population, as determined by total cell counting and intact cell counting, with the success of *L. pneumophila* disinfection according to the standard cultivation method (defined by ISO 11731 [18]). Moreover, we carried out regrowth experiments to evaluate the bacteriostatic or bactericidal effect of each biocide treatment on the microbial population, based on the growth curves determined by the FCM-based approach. In the end, we could show the feasibility of combining total cell counting and intact cell counting to simultaneously analyze biocide effects on cell membranes and DNA and the advantage of this approach in practicability compared to qPCR and cultivation methods.

## Material and methods

### Process water matrix

A lab-scale plastic reactor was used to prepare process water under Biosafety level 2, which simulates the water circulation, water treatments, typical chemical and physical conditions, and bacterial growth in evaporative cooling systems. The industrial corrosion inhibitor ST-DOS K-310 (Schweitzer-Chemie GmbH, Freiberg am Neckar, Germany) and NaOH (≥ 98%, Carl Roth, Karlsruhe, Germany) were added to fully deionized water to simulate the water preparation and to adjust the pH value to 8. NaCl (≥ 99.8%, Carl Roth, Karlsruhe, Germany) was added until the conductivity of the solution reached 1.5 mS/cm. Prepared process water was circulated at 30 °C, which is in the typical range of cooling water in evaporative cooling systems [19], with a sufficient air supply. After at least 14 days, a stable microbial population could naturally grow in the evaporative cooling system simulating system. A sensor system was used to monitor water temperature, pH, and conductivity during bacterial growth to create a stable microbial population. This self-prepared process water was used for device and method characterization, biocide batch experiments, and regrowth experiments.

### Process water containing conditioned *Legionella pneumophila*

A buffered charcoal yeast extract agar plate (Xebios Diagnostics, Düsseldorf, Germany) was streaked with the *L. pneumophila* strain (serogroup 1, subtype Bellingham). Buffered yeast extract liquid growth medium was prepared by adding 5 g Bacto Yeast Extract (Life Technologies, Carlsbad, CA, USA) and one vial *Legionella* BCYE Growth Supplement (VWR Chemicals, Leuven, Belgium) to 500 mL ultrapure water followed by sterile filtration (0.22 µm PES syringe filter, Carl Roth, Karlsruhe, Germany). After 4 days, four colonies were collected and added to 5 mL buffered yeast extract liquid growth medium, followed by 20 h cultivation at 37 °C. The medium containing *L. pneumophila* was 1:1000 diluted in sterile-filtered process water and shaken at 25 °C for 48 h to generate conditioned bacteria. The aim of this conditioning step was to prepare a population with comparable proportions of viable but non-culturable and culturable cells to those found in contaminated process water. The concentration of intact *L. pneumophila* after the conditioning was determined using the detection kits for *L. pneumophila* serogroup 1 (rqmicro AG, Schlieren, Switzerland). In the biocide batch experiments followed by the regrowth experiment, conditioned *L. pneumophila* were spiked in the self-prepared process water to simulate contaminated process water in cooling systems.

### Biocide treatments

Benzalkonium chloride (BAC, ≥ 95.0%, Sigma-Aldrich, St. Louis, MO, USA) was first dissolved in ultrapure water to prepare the stock solution. The free chlorine residual in the NaClO stock solution (12% Cl, stabilized, Carl Roth, Karlsruhe, Germany) was determined according to the colorimetric method using N,N-dialkyl-1,4-phenylenediamine (ISO 7393–2:2017 [20]) on the day the batch experiments were carried out.

At the start of each biocide treatment, the stock solution was added to process water to adjust the initial biocide concentration. During biocide treatment, process water was shaken at 25 °C. 40 mg/L sodium thiosulfate was used as a neutralizer for NaClO, and BAC was quenched by a mixture of 75 mg/L lecithin (≥ 97%, from soybeans, Carl Roth, Karlsruhe, Germany) and 75 mg/L Tween® 80 (purest, Carl Roth, Karlsruhe, Germany). Samples were added to centrifugation tubes with pre-aliquoted neutralizer to stop the biocide treatment at a defined exposure time. In time-dependent effect analysis, the exposure time was set to 1, 5, 15, 30, or 60 min. In the batch experiments, the exposure time was set to 2 h.

### Regrowth experiment

With the evaporative cooling system simulating plant, we could prepare process water without biocide addition for the regrowth experiment. The sterile-filtered process water provided the original growth conditions for the regrowth of naturally occurring microorganisms: original water chemistry, nutrient-poor, unaffected by biocides. After the biocide batch experiments, samples were 1:100 diluted in 150 mL sterile-filtered process water to prepare regrowth samples. Untreated process water was 1:100 diluted in sterile-filtered process water to prepare a positive reference. As a negative control, 150 mL sterile-filtered process water without any spiking was used. Solutions were transferred to sterile shake flasks with sterile membranes that enable gas exchange and then shaken and incubated at 30 °C. During the 8-day regrowth experiment, samples were taken from the flasks, and the changes in TCC and ICC were measured using the flow cytometer rqmicro.COUNT.

### Measurements on FCM rqmicro.COUNT

TCC kits containing SYBR Green and ICC kits containing SYBR Green and propidium iodide (rqmicro AG, Schlieren, Switzerland) were used for total cell counting and intact cell counting measurements, respectively. The reagents and optimized buffers were already provided in lyophilized form in the reaction vials so that only 1 mL of sample needed to be added. Samples were then incubated for 15 min at room temperature. Afterwards, samples were transferred to the FCM cartridge (rqmicro AG, Schlieren, Switzerland) and measured on an rqmicro.COUNT device (rqmicro AG, Schlieren, Switzerland). Eight samples were analyzed simultaneously.

### Cultivation on BCYE

*L. pneumophila* was enumerated using the cultivation method according to ISO 11731:2017 [18]. Samples were directly plated on buffered charcoal yeast extract agar plates with a 0.1 mL or 0.5 mL volume in different dilutions. All plates were incubated at 37 °C for 7 days. Suspicious colonies were cultivated on buffered charcoal yeast extract plates without cysteine (Xebios Diagnostics, Düsseldorf, Germany) to confirm the identity of *L. pneumophila* cells.

### Heterotrophic plate count

For heterotrophic plate count measurements, samples were cultivated according to ISO 6222:1999 [21] on yeast extract agar in different dilutions. Plates were incubated at 22 °C for 36 h or 37 °C for 44 h. After incubation, colonies inside and on top of the agar were counted.

## Results and discussion

### FCM for a time-dependent study of biocide effects

We carried out NaClO (0.2, 0.5, 2, and 5 mg/L free chlorine) and BAC (5, 10, 25 mg/L) treatment for disinfecting naturally occurring microorganisms in process water. The time-dependent biocide effects were analyzed by FCM to test the analytical power of measuring TCC and ICC.

The intercalative binding of SYBR Green is altered when chemical modifications or structure changes in DNA occur [9]. Therefore, the changes in green fluorescence intensity in TCC measurements can reflect DNA damage. We observed that the oxidizing biocide NaClO and the non-oxidizing biocide BAC had distinctively different biocide effects on intracellular DNA measured by TCC.

**Fig. 2:**
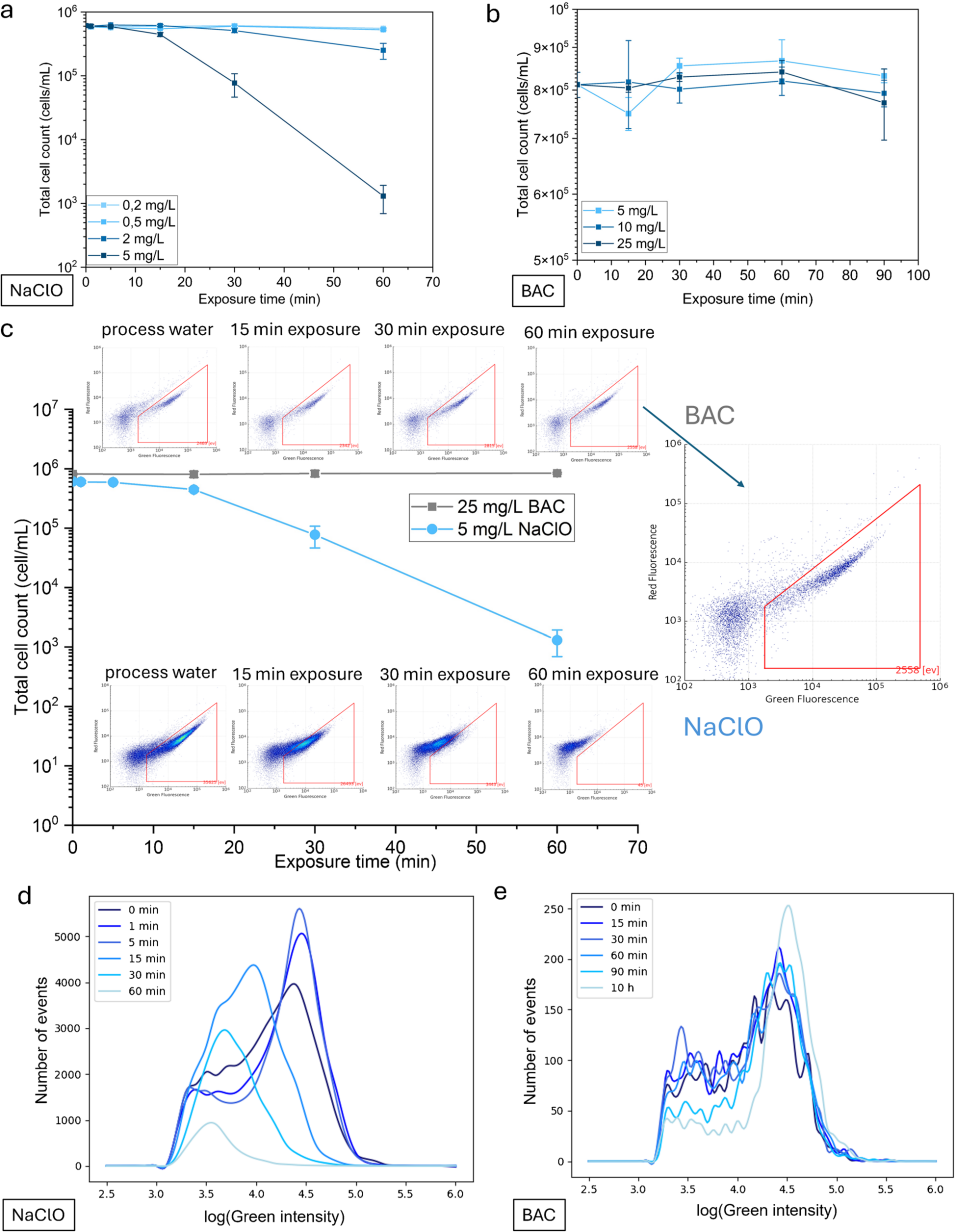
TCC results of NaClO and BAC batch experiments after different exposure times. **a** NaClO, *n* = 3; **b** BAC, *n* = 3; **c** comparison between NaClO and BAC with the corresponding dot plots, without prior dilution for NaClO samples, BAC samples were 1:20 diluted to eliminate the effects of BAC and the neutralizers on the measurement performance, *n* = 3; **d** the changes in green fluorescence intensity distribution during the treatment of 5 mg/L initial free chlorine residual; **e** the changes in green fluorescence intensity distribution during the treatment of 25 mg/L BAC. The red trapezoid in **a** is the default gate for TCC to distinguish between cell signal and background

During NaClO treatment, DNA damage was observed. If we analyze TCC data following the typical analysis method for FCM data, we used the default gate for distinguishing between cell and background signal (red trapezoid in Fig. 2c) and counted the cell signals in the gate. The results of cell counting are presented as TCC in Fig. 2c. The TCC decreased by less than 10% during the first 15 min of exposure to 5 mg/L free chlorine, with a significant reduction observed only after 30 min of exposure (> 30%). However, the examination of the fluorescence intensity reveals an observable reduction in green fluorescence after 15 min of exposure, as indicated by a leftward shift of the cell signal cluster. By plotting cell count in the gate against green fluorescence intensity, we could show that, although the fluorescence intensity of most cells was reduced after 15 min, it remained clearly distinguishable from the background signal (Fig. 2d). As a result, DNA damage after 15 min was significant according to the reduction in fluorescence intensity, but it was not reflected in the TCC. This observation can be explained by the difference in SYBR Green binding. Phe et al. stated that the chlorination effect on isolated double-stranded DNA started with chemical modification of DNA bases, resulting in a chlorine concentration-dependent decrease in green fluorescence intensity following SYBR Green staining. If a high proportion of bases are degraded, DNA is unable to maintain the double-stranded structure due to the breaking of hydrogen bonds. This structure change can alter the binding behavior of SYBR Green and reduce the green fluorescence to a neglectable level [9]. Based on this study of isolated DNA, we would conclude the potential alterations underlying the observed DNA damage. Less severe DNA damage after 15 min of exposure is, therefore, likely due to chemical modifications of bases in double-stranded DNA. After 30 min, more severe DNA damage, which could be breakage of double-stranded DNA into single-stranded DNA, occurred in some cells. Thus, the fluorescence intensity of these cells decreased to background level, leading to the decay in TCC.

Therefore, the decrease in TCC can indicate severe DNA damage. If TCC stays constant, but green fluorescence intensity of some cells is reduced, less severe DNA damage can be assumed. In practice, TCC is an easy-to-use indicator, as it reflects the proportion of cells with severe DNA damage by a simple number. If further information on less severe DNA damage is needed, cells with reduced green fluorescence intensity can be counted. Our results also question the methodology used in previous studies, which has relied on the proportion of cells with reduced fluorescence intensity [8, 22].

In comparison, BAC had no significant effect on intracellular DNA. As shown in Fig. 2e, green fluorescence intensity showed no significant reduction during the 10 h of incubation, indicating that DNA staining by SYBR Green was not affected by BAC treatment. This result is consistent with previous studies on BAC [5]. TCC was constant at 8 × 10^5^ cells/mL during the 60-min incubation at the highest BAC concentration (25 mg/L), suggesting that TCC can serve as a specific indicator of DNA damage.

**Fig. 3:**
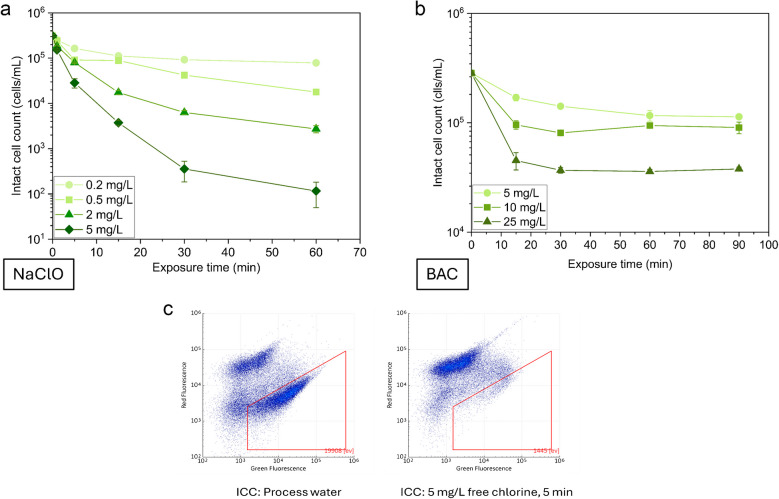
ICC results of NaClO and BAC batch experiments after different exposure times. **a** NaClO, *n* = 3; **b** BAC, *n* = 3; **c** representative dot plots to demonstrate the fluorescence intensity shift in ICC plots during biocide treatments. The red trapezoid in **c** is the default gate for ICC to distinguish the signal of intact cells from the signal of damaged cells and background

Intact cell counting uses SYBR Green and propidium iodide to determine ICC, representing the count of cells with intact cell membranes and stainable DNA. As expected, ICC decreased with increasing biocide concentrations and exposure time (Fig. 3a and b). For all three concentrations, BAC exhibited a rapider effect on cell membranes compared to NaClO. At the highest concentrations, 5 mg/L NaClO caused a much higher reduction in ICC (3.4-log) compared to 25 mg/L BAC (1-log), indicating a strong effect of NaClO on cell membranes. These results confirm that ICC by FCM provides a promising method for analyzing biocide effects on cell membrane integrity.

**Fig. 4:**
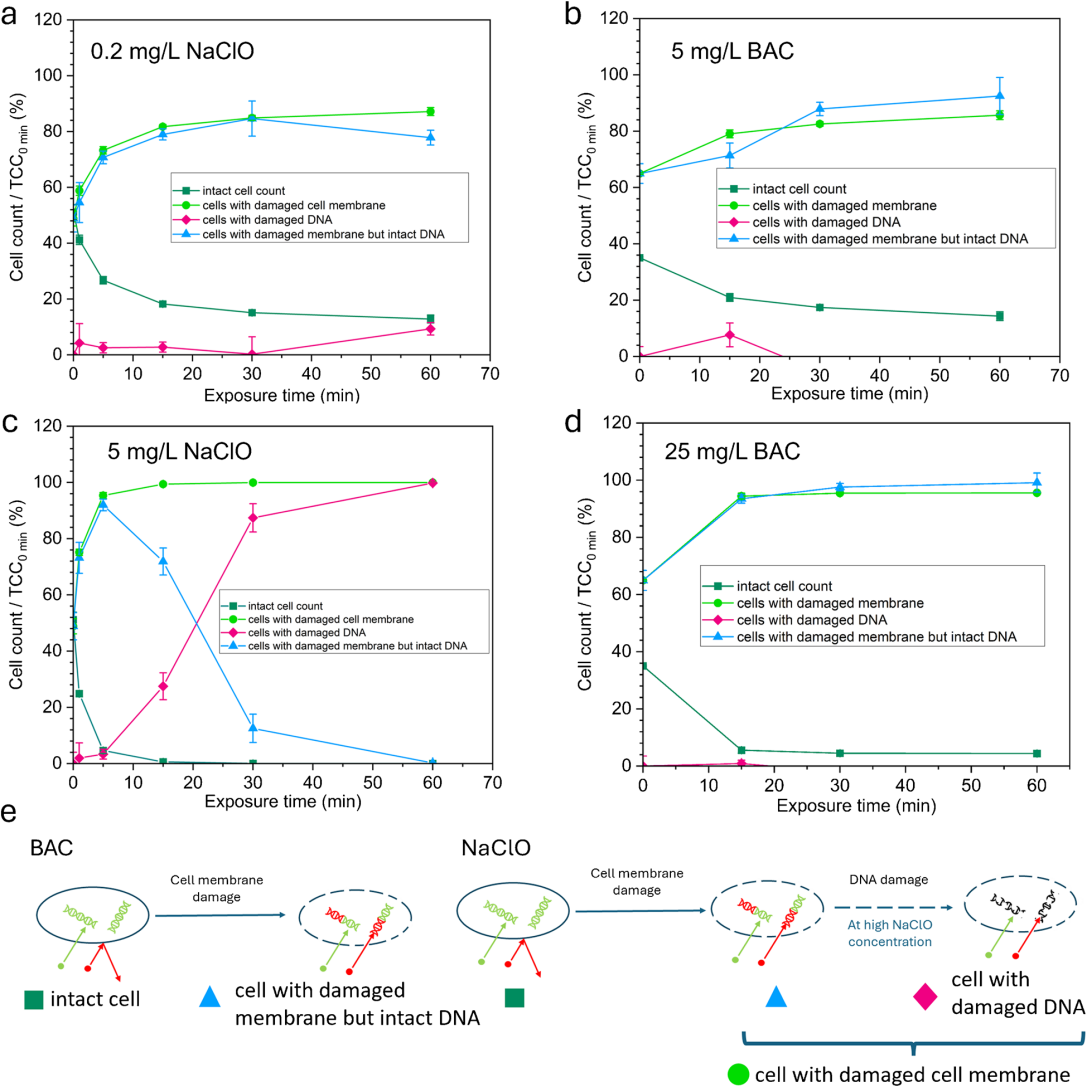
Changes in the proportions of different states of cells during different treatments. **a** 0.2 mg/L initial free chlorine residual; **b** 5 mg/L BAC; **c** 5 mg/L initial free chlorine residual; **d** 25 mg/L BAC; **e** illustration of different cells presented in **a**–**d**; *n* = 3 in **a**–**d**

Based on TCC and ICC results, we could demonstrate the time-dependent biocide effects of NaClO and BAC. We calculated the concentrations of cells in different states from TCC and ICC using Eqs. 1–3 and illustrate the results in Fig. 4.

1 $${\mathrm{TCC}}_{0}-\mathrm{ICC}=\text{count of cells with damaged cell membrane}$$

2 $$\mathrm{TCC}-\mathrm{ICC}=\text{count of cells with damaged cell membrane},\text{ but intact DNA}$$

3 $${\mathrm{TCC}}_{0}-\mathrm{TCC}=\text{count of cells with damaged DNA}$$

TCC_0_ refers to the TCC before biocide treatment.

As shown in Fig. 4a and c, different concentrations of NaClO affected different cell components. At the lowest initial concentration of 0.2 mg/L, no DNA damage was observed (Fig. 4a), and the reduction level in ICC after 60 min was much lower (0.6-log). The low free chlorine residual after treatment (under 0.1 mg/L, determined by the colorimetric method according to ISO 7393–2:2017) could be the reason for the absence of DNA damage. In contrast, 5 mg/L initial free chlorine led to DNA damage (Fig. 4c) and retained a high free chlorine residual of 3 mg/L after a reaction time of 60 min. During the treatment with 5 mg/L initial free chlorine, the ICC first dropped sharply to 2%, showing that most cell membranes were damaged; then the count of cells with damaged DNA started to significantly increase (Fig. 4c). A likely explanation is that ionized ^−^OCl, which dominates the HOCl-^−^OCl equilibrium under the alkaline pH value (pH = 8) in process water, has a low cell membrane permeability [16]. Once the cell membrane integrity was compromised, ^−^OCl could enter more freely, leading to a higher concentration of free chlorine in cells that can affect DNA.

Unlike NaClO, as shown in Fig. 4b and d, BAC showed lytic, but no genotoxic effects at both the lowest and highest concentration, which was consistent with the previous studies [5]. The effects of the lowest and highest BAC concentrations differed only in the extent of ICC reduction (0.41-log for 5 mg/L and 0.94-log for 25 mg/L).

To sum up, we can conclude that a combination of TCC and ICC by the FCM rqmicro.COUNT is a reliable approach for analyzing damage in DNA and cell membranes of a complex microbial population and for distinguishing the modes of action of the oxidizing biocide NaClO and the non-oxidizing biocide BAC. The effects of NaClO can be separated into two stages. In the first stage, NaClO damaged cell membranes so that it could enter the cell through the broken membrane. In the second stage, NaClO damaged and even destroyed the DNA inside the cells. In comparison, BAC did not cause DNA damage and affected cell membrane integrity to a smaller extent, even at the highest concentration in the experiment.

### Apply FCM for biocide efficiency analysis in process water contaminated by Legionella pneumophila

We carried out two biocide batch experiments to confirm that total cell counting and intact cell counting by the FCM rqmicro.COUNT could be applied for process water contaminated by *L. pneumophila*, the pathogen of concern in evaporative cooling systems. Considering the practical purposes of biocide application in evaporative cooling systems, we evaluated the biocide efficiency from two different perspectives: (i) could the biocide treatment eliminate the microbiological risk caused by *L. pneumophila*? (ii) could biocide treatment inhibit microbial regrowth (bacteriostatic effect) afterwards or even prevent microorganisms from regrowth (bactericidal effect)? For the former, we conducted the standard cultivation test method for *L. pneumophila* according to ISO 11731:2017 [18] parallel to TCC and ICC measurements before and after biocide treatment. For the latter, we carried out a regrowth experiment based on TCC and ICC to analyze the bacteriostatic versus bactericidal effects.

We spiked aliquots into prepared process water with naturally occurring microorganisms to simulate contaminated process water in evaporative cooling systems. The concentrations of the spikes were adjusted to prepare a batch of process water that was less contaminated with *L. pneumophila* (batch 1) and a batch of process water more contaminated with *L. pneumophila* (batch 2), where the naturally occurring microbiome—which would contribute also to TCC and ICC—was otherwise identical. The information on the two batches of biocide experiments is presented in Table 1. According to the German standard VDI 2047 [23] and the Australian standard AS/NZS 3666.3:2000 [24], numbers of 1000 CFU/100 mL (i.e., 10 CFU/mL) and 10000 CFU/100 mL (i.e., 100 CFU/ml) are two threshold values for determining necessary actions for *L. pneumophila* disinfection. As shown in Table 1, the *L. pneumophila* concentration in batch 1 was between the two threshold values, whereas the one in batch 2 was above the higher threshold value. We analyzed the TCC and ICC to quantify cells, including culturable and viable but non-culturable *L. pneumophila* as well as other naturally occurring microorganisms. As the cultivation method according to ISO 11731 can only detect culturable *Legionella* spp., the TCC and ICC, which are unspecific indicators, are not well correlated with the results according to ISO 11731. Nevertheless, the changes in TCC and ICC can reflect the biocide effects on the microbial population, which could correlate with the biocide effects on *L. pneumophila*. Therefore, we carried out biocide tests on the two batches of process water with different levels of *L. pneumophila* contamination and used the two biocides tested in the time-dependent study. The results are shown in Table 1.

**Table 1:** Information on biocide batch experiments. Two batches of process water with the same temperature, pH, and conductivity but different initial cell counts were used. The exposure time was 2 h for all treatments

	Process water batch 1 (less contaminated)				Process water batch 2 (more contaminated)			
TCC per mL before^a^	(9.0 ± 0.3) × 10^5^				(1.7 ± 0.07) × 10^6^			
ICC per mL before^a^	(2.5 ± 0.1) × 10^5^				(9.7 ± 0.2) × 10^5^			
Culturable *L. pneumophila* before in CFU/mL^b^	43				1060			
Biocide	NaClO		BAC		NaClO		BAC	
Biocide concentration in mg/L	0.5	2.5	5	25	0.2	1	5	25
TCC per mL after^a^	(6.3 ± 1.0) × 10^5^	(6.4 ± 2.3) × 10^2^	(1.0 ± 0.03) × 10^6^	(1.0 ± 0.4) × 10^6^	(1.7 ± 0.01) × 10^6^	(1.7 ± 0.04) × 10^6^	(1.7 ± 0.05) × 10^6^	(1.7 ± 0.07) × 10^6^
ICC per mL after^a^	(1.8 ± 0.1) × 10^4^	(1.6 ± 0.8) × 10^2^	(1.4 ± 0.02) × 10^5^	(5.3 ± 0.3) × 10^4^	(7.2 ± 0.2) × 10^5^	(6.2 ± 0.3) × 10^4^	(1.4 ± 0.1) × 10^5^	(5.9 ± 0.3) × 10^4^
ICC_after_/ICC_before_	(7.1 ± 0.5)%	(0.06 ± 0.03)%	(58.4 ± 2.6)%	(21.0 ± 1.5)%	(73.9 ± 2.0)%	(6.3 ± 0.4)%	(14.3 ± 1.0)%	(6.0 ± 0.3)%
TCC_after_/TCC_before_	(69.7 ± 11.0)%	(0.07 ± 0.03)%	(113.2 ± 2.8)%	(114.3 ± 4.4)%	(97.9 ± 4.2)%	(100.2 ± 4.8)%	(96.4 ± 5.1)%	(101.1 ± 6.0)%
Culturable *L. pneumophila* after in CFU/mL^b^	0	0	3	0	530	0	0	0

^a^ “Before” and “after” refer to the cell counts before or after biocide treatments, *n* = 3

^b^*L. pneumophila* was detected according to ISO 11731:2017 [18]. Measurements were carried out before and after biocide treatment

We could evaluate damage in cell membranes and intracellular DNA using the changes in ICC and TCC, which were presented as ICC_after_/ICC_before_ and TCC_after_/TCC_before_, respectively. After the treatment of 0.5 mg/L NaClO, the process water batch 1 underwent a 30% decrease in TCC. Enhancing the treatment concentration to 2.5 mg/L, the TCC in batch 1 further reduced substantially to less than 1%. These significant decreases in TCC were only observed after NaClO treatments in batch 1, accompanied by high standard deviations ((69.7 ± 11.0)% for 0.5 mg/L and (0.07 ± 0.03)% for 2.5 mg/L), which were consistent with the DNA damaging effect of NaClO determined in previous time-dependent biocide experiments. For the 2.5 mg/L NaClO treatment in batch 1, TCC and ICC results were no more precise because DNA was too severely damaged. For batch 2, we reduced the NaClO dosages to investigate whether the biocide effect on DNA would differ. The results showed that the changes in TCC after NaClO treatments were within ± 4%, indicating no DNA damage. The possible explanation is that the higher concentration of cells in batch 2 consumed NaClO before ^−^OCl could permeate the compromised cell membranes and react with DNA. BAC treatments led to no decrease in the TCC. In batch 2, the changes in TCC after BAC treatments were within ± 4%. Therefore. No DNA damage was determined after BAC treatments, which corresponded to the results of time-dependent biocide experiments.

Biocide effects on cell membranes were observed after both NaClO and BAC treatments. Except for the 0.2 mg/L treatment in batch 2, NaClO treatments damaged more than 90% of intact cell membranes. For BAC treatments, the cell membrane damaging effect was considerably enhanced at an elevated concentration (5 to 25 mg/L) in the same batch. Comparing the lower ICC_after_/ICC_before_ results of batch 1 to batch 2, a larger proportion of intact cell membranes was damaged by the same concentration of BAC (5 or 25 mg/L) in batch 2, which had a higher load of *L. pneumophila* and other microorganisms. A possible explanation is that some species of the naturally occurring microbiome exhibited resistance against BAC and retained intact cell membranes after BAC treatment. For instance, McDonnell and Russell concluded that spore-forming organisms and mycobacteria demonstrated intrinsic resistance to quaternary ammonium (including BAC) due to their complex cell wall structures [25]. The remaining intact cells likely represented BAC-resistant microorganisms that naturally occurred in the process water and were present at the same concentration in both batches. Batch 2 had a higher initial ICC, resulting in a lower ICC_after_/ICC_before_ ratio because the ICC_after_ value was similar between the two batches.

To summarize, we could analyze biocide effects on cell membranes and intracellular DNA in process water contaminated by *L. pneumophila*. The next step is to investigate the correlations of the factor ICC_after_/ICC_before_ with the success of *L. pneumophila* disinfection. As shown in Table 1, culturable *L. pneumophila* were only detected after two treatments: 5 mg/L BAC in batch 1 and 0.2 mg/L NaClO in batch 2. The factor ICC_after_/ICC_before_ was also exceptionally high in these two treatments ((58.4 ± 2.6)% and (73.9 ± 2.0)%), indicating low biocide efficiency regarding membrane damaging effects. All other treatments led to ICC_after_/ICC_before_ lower than 21% and total removal of culturable *L. pneumophila* according to ISO 11731:2017, which indicated successful disinfection. As a result, a low factor ICC_after_/ICC_before_ (< (21.0 ± 1.5)% in this work) could be linked to the success of *L. pneumophila* disinfection in our experiments.

### Regrowth experiment and FCM-based regrowth curve

We carried out a regrowth experiment after each treatment to determine the bacteriostatic and bactericidal effects of biocide treatments. We used this sterile-filtered and untreated process water to dilute (1:100) the treated samples (batch 1 and batch 2 of Table 1, see above) to eliminate the effects of biocide residues and excessive neutralizers. Alongside each batch of process water, we introduced the untreated process water with naturally occurring microorganisms and spiked *L. pneumophila* as a positive control. Sterile-filtered process water was used as a negative control.

**Fig. 5:**
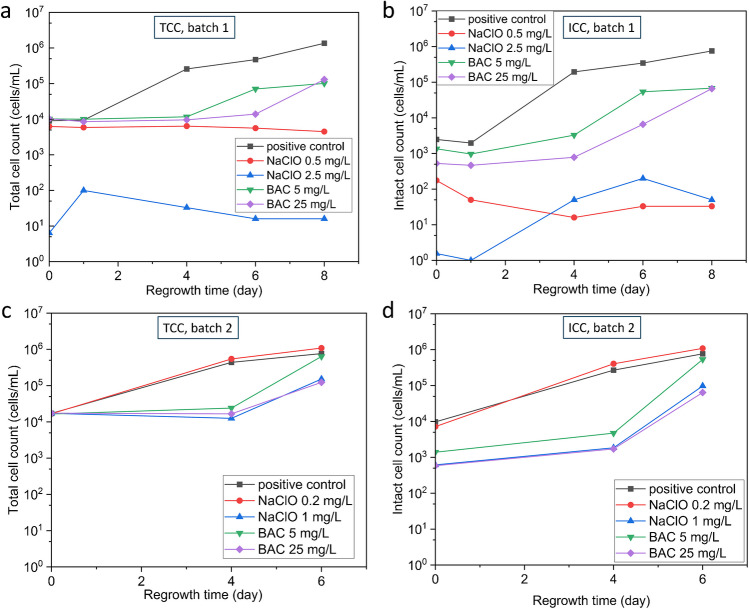
Results of regrowth experiments. **a** TCC changes during the regrowth after treatments in batch 1; **b** ICC changes during the regrowth after treatments in batch 1; **c** TCC changes during the regrowth after treatments in batch 2; **d** ICC changes during the regrowth after treatments in batch 2. Positive control refers to untreated process water with spiked *L. pneumophila*. Sterile-filtered process water was used as negative control, and no regrowth was observed in negative control (TCC and ICC < 35 cells/mL)

Figure 5 shows the changes in TCC and ICC during the regrowth experiments. The positive control was in the exponential growth phase until the end, so the data could provide a reasonable reference for the regrowth experiments. The initial ICC and TCC of each regrowth experiment represented the state of the (diluted) microbial population after the corresponding biocide treatment. Figure 5 a and b show that the regrowth of microorganisms in batch 1 treated with 5 and 25 mg/L BAC was considerably inhibited after the treatments. The growth curves after these two treatments had a longer lag until the start of the exponential growth phase than the positive control curve. The growth curve of 5 mg/L BAC treatment increased at a faster rate than the curve of 25 mg/L in Fig. 5b and had a shorter lag phase in Fig. 5a. As a result, we could conclude that the 5 and 25 mg/L BAC treatment in batch 1 had bacteriostatic effects on the microbial population. The bacteriostatic effect of 25 mg/L BAC treatment was more substantial than that of 5 mg/L BAC. After the 0.5 and 2.5 mg/L NaClO treatment, the ICC remained below the lower limit of the linear detection range of the method (see Electronic Supplementary Material Fig. S1) in Fig. 5b. Therefore, after treatment with 0.5 and 2.5 mg/L NaClO, the microbial population failed to regrow in the nutrient-poor environment within 8 days, indicating bactericidal effects.

In batch 2, no decrease in TCC was observed. This is consistent with the trends in Fig. 5c and d, in which all curves significantly increase. The ranking of increase rates in Fig. 5c and d followed the trend of ICC_after_/ICC_before_. The 0.2 mg/L NaClO treatment had an ICC_after_/ICC_before_ value of (73.9 ± 2.0)%, which was much higher than in other treatments. The corresponding regrowth curve closely overlaps with the positive control curve in Fig. 5c and d, indicating that the minimal biocide effect on cell membranes was related to an essentially non-existing bacteriostatic effect. The 5 mg/L BAC curve exhibits the second fastest rate of increase among all curves of treated samples in Fig. 5c and b, corresponding to an ICC_after_/ICC_before_ value of (58.4 ± 2.6) %. It is followed by the trend for 1 mg/L NaClO and 25 mg/L BAC in Fig. 5c and d, which shared a similar ICC_after_/ICC_before_ value (6.3% and 6.0%).

To sum up, the TCC_after_/TCC_before_ and ICC_after_/ICC_before_ ratios, which reflect changes in the TCC and ICC, serve as indicators helping us to distinguish between the bacteriostatic and bactericidal effects of NaClO and BAC. NaClO exhibited bactericidal effects when DNA damage, indicated by a significant decrease in the TCC, occurred. In contrast, it showed strong bacteriostatic effects when a high proportion of cell membranes was damaged without DNA damage, as indicated by a significant decrease in ICC_after_/ICC_before_ ratio and no decrease in the TCC. BAC showed bacteriostatic effects when the ICC significantly decreased after BAC treatments. No bactericidal effects were observed after BAC treatments, consistent with the absence of a decrease in the TCC. When addressing the longevity of the treatment, our experiments show that microorganisms proliferated already only 4 days after all the BAC treatments, where two BAC treatments in the same batch showed minor differences in the regrowth rate. Consequently, our results indicate that NaClO treatments are more promising and that they can be optimized with the aid of a flanking analysis of TCC and ICC to achieve the strongest bacteriostatic or even bactericidal effects. In contrast, BAC treatments would have to be repeated more frequently at lower concentrations to enhance microbial growth inhibition while minimizing biocide dosage.

In the final step, we compared the TCC and ICC results with those from the conventional cultivation method for analyzing waterborne microbial populations, namely the heterotrophic plate count method, to assess the competitiveness of the FCM-based method for distinguishing bactericidal from bacteriostatic effects. The heterotrophic plate count method is a standard cultivation method defined by ISO 6222:1999 [21] for enumerating culturable microorganisms in drinking water and wastewater, which is also applied for detecting general colony counts in evaporative cooling systems (VDI 2047 [23]).

We conducted heterotrophic plate count measurements parallel to the total cell counting and intact cell counting by FCM to analyze the biocide treatments. Table 2 shows that the heterotrophic plate count method provided zero colony results for 2.5 mg/L NaClO in batch 1, 1 mg/L NaClO in batch 2, and 25 mg/L BAC in both batches. However, these treatments exhibited different effects. Only 2.5 mg/L NaClO in batch 1 had bactericidal effects. In contrast, 0.5 mg/L NaClO in batch 1 showed bactericidal effects in the regrowth experiment. However, heterotrophic plate count 22 °C detected colonies after the treatment. It suggests that some microorganisms may be culturable on the medium but could not grow in process water. The cultivability on the nutrient-rich growth medium cannot represent the ability of regrowth in the nutrient-poor matrix. Moreover, NaClO 0.2 mg/L in batch 2 showed a negligible bacteriostatic effect in the regrowth experiment but sharply reduced heterotrophic plate count 37 °C and 22 °C (Table 2). For this treatment, the biocide efficiency could be strongly overestimated according to the heterotrophic plate count method. To conclude, the cultivation-independent analysis method measuring TCC and ICC by FCM provided results that were significantly better correlated with estimations of bacteriostatic versus bactericidal effects than the cultivation results provided by the heterotrophic plate count method.

**Table 2:** Comparison of total cell counting and intact cell counting by FCM with the heterotrophic plate count method according to ISO 6222:1999 [21] for analyzing biocide treatments. *HPC* refers to the heterotrophic plate count in this table

Treatment		ICC_after_/ICC_before_	TCC_after_/TCC_before_	HPC_37 °C, after_/HPC_37 °C, before_	HPC_22 °C, after_/HPC_22 °C, before_	Effect
Batch 1	NaClO 0.5 mg/L	(7.1 ± 0.5)%	(69.7 ± 11.0)%	0%	5.7%	Bactericidal
	NaClO 2.5 mg/L	(0.06 ± 0.03)%	(0.07 ± 0.03)%	0%	0%	Bactericidal
	BAC 5 mg/L	(58.4 ± 2.6)%	(113.2 ± 2.8)%	4.0%	40.3%	Bacteriostatic
	BAC 25 mg/L	(21.0 ± 1.5)%	(114.3 ± 4.4)%	0%	0%	Bacteriostatic
Batch 2	NaClO 0.2 mg/L	(73.9 ± 2.0)%	(97.9 ± 4.2)%	6.0%	9.8%	No effect
	NaClO 1 mg/L	(6.3 ± 0.4)%	(100.2 ± 4.8)%	0%	0%	Bacteriostatic
	BAC 5 mg/L	(14.3 ± 1.0)%	(96.4 ± 5.1)%	0.9%	0%	Bacteriostatic
	BAC 25 mg/L	(6.0 ± 0.3)%	(101.1 ± 6.0)%	0%	0%	Bacteriostatic

In summary, total cell counting and intact cell counting by FCM could be applied for biocide analysis in process water contaminated by *L. pneumophila*. The TCC and ICC results of treatments in batch 1 and 2 were in excellent agreement with the modes of action of NaClO and BAC determined in time-dependent biocide experiments. Using this cultivation-independent method, we also successfully monitored the regrowth of microorganisms after biocide treatments and determined the bacteriostatic versus bactericidal effects of the treatments. In our experiments, a low value of the factor ICC_after_/ICC_before_ was a dependable indicator for the success of *L. pneumophila* disinfection according to the standard method. Moreover, a low ICC_after_/ICC_before_ value in the treatment was also correlated with bacteriostatic effects in the corresponding regrowth experiment. Most importantly, our data suggest that when the TCC drops after a NaClO treatment, bactericidal effects can be expected.

## Conclusion

This work aimed to exploit the potential of total cell counting and intact cell counting by FCM as an effect-based and rapid method in biocide efficiency evaluation in evaporative cooling systems. In time-dependent biocide experiments, we showed that it is feasible to use this method for the simultaneous analysis of damage in cell membranes and intracellular DNA caused by the oxidizing biocide NaClO and the non-oxidizing BAC. We confirmed the hypothesis that severe DNA damage, like structure changes from double-stranded DNA to single-stranded DNA, was reflected by the reduction in TCC. In addition, the reduction in green fluorescence intensity to a smaller detectable level in TCC measurements could indicate the presence of less severe DNA damage. According to the effect of DNA and membrane damage, and consistent with previous studies, we determined the different modes of action of NaClO and BAC. Subsequently, we applied the FCM-based approach for biocide analysis in process water with two different batches of *L. pneumophila* contamination in different concentrations. After treatment, we conducted regrowth experiments and determined the bacteriostatic versus bactericidal effects of the treatments using total cell counting and intact cell counting by FCM. Based on the results, we could suggest that the biocide effects, which severely damaged cell membranes, were related to bacteriostatic effects on the microbial population. For NaClO, bactericidal effects could be indicated by the occurrence of DNA damage, as reflected in the decrease in TCC. Moreover, culturable *L. pneumophila* were eradicated when the ICC significantly decreased. It indicates that the biocide effects on *L. pneumophila* and on the entire microbial population were consistent, suggesting that *L. pneumophila* did not exhibit any additional resistance against these two biocides. Hence, even though TCC and ICC are non-specific analytical methods, these results show that the changes in the TCC and ICC are reliable proxies for the success of *L. pneumophila* disinfection.

With these results, we intended to advance total cell counting and intact cell counting by FCM toward applications as an operational analytical tool. In practice, it can be applied as a rapid method for biocide efficiency analysis, which provides same-day results for decision-making in biocide treatment. Our data showed that the drop in TCC and ICC_after_/ICC_before_ could provide dependable proxies for biocide efficiency assessment regarding both pathogen disinfection and microbial growth inhibition. In contrast, the standard cultivation method for general colony counts according to ISO 6222 randomly overestimated or underestimated biocide efficiency regarding the inhibition of microbial growth.

This rapid, affordable, and on-site approach has the potential to facilitate the adoption of daily microbiological analysis, thereby enabling a need-oriented biocide application in evaporative cooling systems. According to regrowth experiments, we suggested that stock NaClO dosage at relatively high concentrations is beneficial for strong bacteriostatic or even bactericidal effects and regular BAC dosage at relatively low concentrations for effective microbial growth control according to the regrowth experiments. As an outlook, these suggestions can be further tested in actual evaporative cooling systems under monitoring with total cell counting and intact cell counting by FCM to investigate if they can help optimize biocide efficiency with minimized biocide dosage. For every individual cooling system, it is also possible to use this method to monitor the regrowth after different treatments and customize a dosage strategy, similar to our regrowth experiments. Furthermore, this approach offers an innovative solution for biocide analysis by employing non-specific proxies to estimate disinfection efficiency against specific pathogens. It is applicable not only to evaporative cooling systems but also to other engineered water systems where biocides are routinely dosed, and it can contribute to more sustainable biocide usage.

## Supplementary Information

Below is the link to the electronic supplementary material.

Supplementary file1 (DOCX 46 KB)

## Data Availability

The data supporting the findings of this study are available within the article and its supplementary information. Further inquiries can be directed to the corresponding author.
