# Supplementary material for: Flow cytometry for rapid analysis of bacteriostatic versus bactericidal effects in Legionella pneumophila disinfection
Source: Anal Bioanal Chem. 2025 Aug 14;418(16):5083–96. doi: 10.1007/s00216-025-06055-z (PMC13424643; doi:10.1007/s00216-025-06055-z)
Supplement: Supplementary file 1 — Supplementary file1 (DOCX 46 KB) [file 216_2025_6055_MOESM1_ESM.docx]

Supplementary Information

Flow cytometry for rapid analysis of bacteriostatic versus bactericidal effects in *Legionella pneumophila* disinfection

Yiao Liang^a^, Lena Heining^a^, Martin Elsner^a^, Michael Seidel^a^*

^a^Institute of Water Chemistry, Chair of Analytical Chemistry and Water Chemistry, School of Natural Sciences, Technical University of Munich, Lichtenbergstraße 4, 85748 Garching, Germany

**Linear detection range**

**Fig. S1** Results of the dilution experiments with process water. The naturally occurring microbial population was diluted in sterile filtered process water and measured.

As shown in Fig. S1, TCC and ICC measurements exhibited good linearity within the detection range of 89–4.6 x 10^5^ cells/mL and 72–3.0 x 10^5^ cells/mL with a R^2^ value of 0.9993 and 0.9998, respectively. When the cell count was reduced to lower than 100 cells/mL, the measurement results no longer followed a linear response. Moreover, the standard deviations of these measured values were significantly higher than those within the linear detection range, indicating a decline in measurement precision.

**Limit of detection**

Blank measurements were carried out by analyzing 20 samples of sterile-filtered process water (0.22 µm PES syringe filter, Carl Roth, Karlsruhe, Germany). The limit of detection, based on the lowest signal above background, was determined by adding three times the standard deviation to the mean value of the blank. The determined limit of detection for TCC measurements is 96 cells/mL, and for ICC measurements, it is 32 cells/mL.

**Intra-assay variability**

Different samples were used to assess intra-assay variability: process water samples collected on five different days, a 1:10 diluted process water sample, and three sterile-filtered process water samples spiked with *Legionella pneumophila* at concentrations of 10⁴, 10⁵, and 10⁶ cells/mL level, respectively. Intra-assay variability was calculated by dividing the standard deviation by the mean value of triplicate measurements for each sample. The calculated intra-assay variability for both TCC and ICC was less than 8%.
